# Cellular Senescence in Livers from Children with End Stage Liver Disease

**DOI:** 10.1371/journal.pone.0010231

**Published:** 2010-04-21

**Authors:** Gabriela Gutierrez-Reyes, Maria del Carmen Garcia de Leon, Gustavo Varela-Fascinetto, Pedro Valencia, Ruy Pérez Tamayo, Claudia Gonzalez Rosado, Blanca Farfan Labonne, Norma Morales Rochilin, Rosalinda Martinez Garcia, Jonathan Aguirre Valadez, Gabriela Togno Latour, Dana Lau Corona, Guillermo Robles Diaz, Albert Zlotnik, David Kershenobich

**Affiliations:** 1 Department of Experimental Medicine, School of Medicine, Universidad Nacional Autónoma de México (UNAM), Mexico City, Mexico; 2 Department of Surgery and Pathology, Hospital Infantil de México Federico Gomez, Mexico City, Mexico; 3 University of California Irvine, Irvine, California, United States of America; Roswell Park Cancer Institute, United States of America

## Abstract

**Background:**

Senescent cells occur in adults with cirrhotic livers independent of the etiology. Aim: Investigate the presence rate of cellular senescence and expression of cell cycle check points in livers from children with end stage disease.

**Methodology/Principal Findings:**

Livers of five children aged three years or less undergoing liver transplantation due to tyrosinemia (n = 1), biliary atresia (n = 2), or fulminant hepatitis (n = 2) were analyzed for senescence associated β-galactosidase (SA-βgal) activity and p16INK4a, p21cip1 and p53. All livers displayed positive cellular staining for SA-βgal in the canals of Hering and interlobular biliary ducts. In the presence of cirrhosis (3/5 cases) SA-βgal was found at the cholangioles and hepatocytes surrounding the regenerative nodules. Children with fulminant hepatic failure without cirrhosis had significant ductular transformation with intense SA-βgal activity. No SA-βgal activity was evident in the fibrous septa. Staining for p53 had a similar distribution to that observed for SA-βgal. Staining for p16^INK4a^ and p21^cip1^ was positive in the explanted liver of the patient with tyrosinemia, in the hepatocytes, the canals of Hering, cholangioles and interlobular bile ducts. In the livers with fulminant hepatitis, p21^cip1^ staining occurred in the areas of ductular transformation and in the interlobular bile ducts.

**Conclusions/Significance:**

Cellular senescence in livers of children with end stage disease is associated with damage rather than corresponding to an age dependent phenomenon. Further studies are needed to support the hypothesis that these senescence markers correlate with disease progression.

## Introduction

In contrast to other types of cellular responses such as necrosis or apoptosis, the role of cellular senescence in the living organism is still not well understood. Senescence was originally described in human fibroblasts as a terminal non dividing stage reached after many cell divisions in cultures [1]Senescent cells remain alive, metabolically active and resistant to apoptotic death but are arrested in the G1 phase of the cell cycle, are resistant to growth factor stimulation and show common biochemical markers, such as expression of an SA-βgal enzyme [2]–[3]. While senescence has been characterized primarily in cultured cells, there is also evidence that it occurs in vivo [4]–[6].

An increase in liver cells staining positive for SA-βgal has been demonstrated in adults with cirrhotic livers independent of the etiology, including chronic viral hepatitis, autoimmune liver disease, primary biliary cirrhosis and chronic alcoholic liver disease; in these situations, chronic liver damage has been related to oxidative stress or telomere shortening, eventually culminating in replicative senescence [6]–[9]. Senescence has also been described to be associated to the progression of fibrosis in hepatitis C virus recurrence after liver transplantation, where a correlation has been observed between ischemic necrosis and replicative senescence on the revascularization biopsy, suggesting that livers containing senescent cells may be more sensitive to ischemia [10].

The genes associated with senescence have for the most part been tumor suppressor genes believed to be involved in the underlying mechanisms of replicative and stress-induced senescence [11]–[15]. p53 functions as a central integration point for various signaling pathways of senescence and inhibits cell division primarily through a p21^cip1^ pathway [16]–[18]. The phosphorylated p53 upregulates transcription of the target gene p21^cip1^, which in turn activates pRb through inhibition of a cyclin-dependent kinase (Cdk) complex [19]. The activated pRb inhibits the transcription of E2F target genes, which are required for cell cycle progression. Another Cdk inhibitor, p16^INK4a^, which also activates pRb, accumulates in senescent cells and is considered essential in stress-induced senescence [13], [14], [20], [21], [22].

In this study, we report the presence of senescence biomarkers, including SA-βgal activity and the expression of p53, p21^cip1^, and p16^INK4a^, in liver from children with end stage liver disease that required transplantation.

## Results

### Analysis of the explanted livers

The liver of the patient with cirrhosis secondary to tyrosinemia (case 1) exhibited, on cut sections, multiple nodules that varied in size from 0.2 to 2.0 cm. The hepatocytes differed in size and shape, their cytoplasm contained lipid droplets, granular material and numerous mitochondria. Some had a dysplastic appearance with atypical nuclei. Fibrous septa of variable size were present; these hepatocytes had a variable immunohistochemical expression of nuclear proliferation factors and fetoproteins (data not shown). The dysplastic hepatocytes were not present in the initial diagnostic biopsy performed two years ago.

In the patients with extrahepatic biliary atresia (cases 2, 3) histology revealed the presence of micronodular cirrhosis. Active fibro-inflammatory septa with cholangiolar proliferation, mild lymphocytic infiltrate and cholestasis were found in case 2, while fibrous septa without bile ducts were observed in case 3. When compared with the initial diagnostic biopsies, the explanted livers of the two patients showed that fibrosis had progressed and both had the typical changes of biliary cirrhosis.

In the children with fulminant hepatic failure (cases 4, 5) the liver had severe gross deformation, large areas of necrosis that were intermingled with nodules of residual hepatic parenchyma; huge necrotic zones were observed exhibiting a dense inflammatory infiltrate composed mainly of mononuclear cells. Immunohistochemical studies revealed, in case 4, a predominant B cell response with equal number of *Kappa* and *Lambda* cells, while in case 5 the cellular infiltrate was predominantly composed of T lymphocytes (CD3, CD45RO; data not shown).

Both livers exhibited significant ductular transformation.

### SA-βgal activity

**Figure 1:**
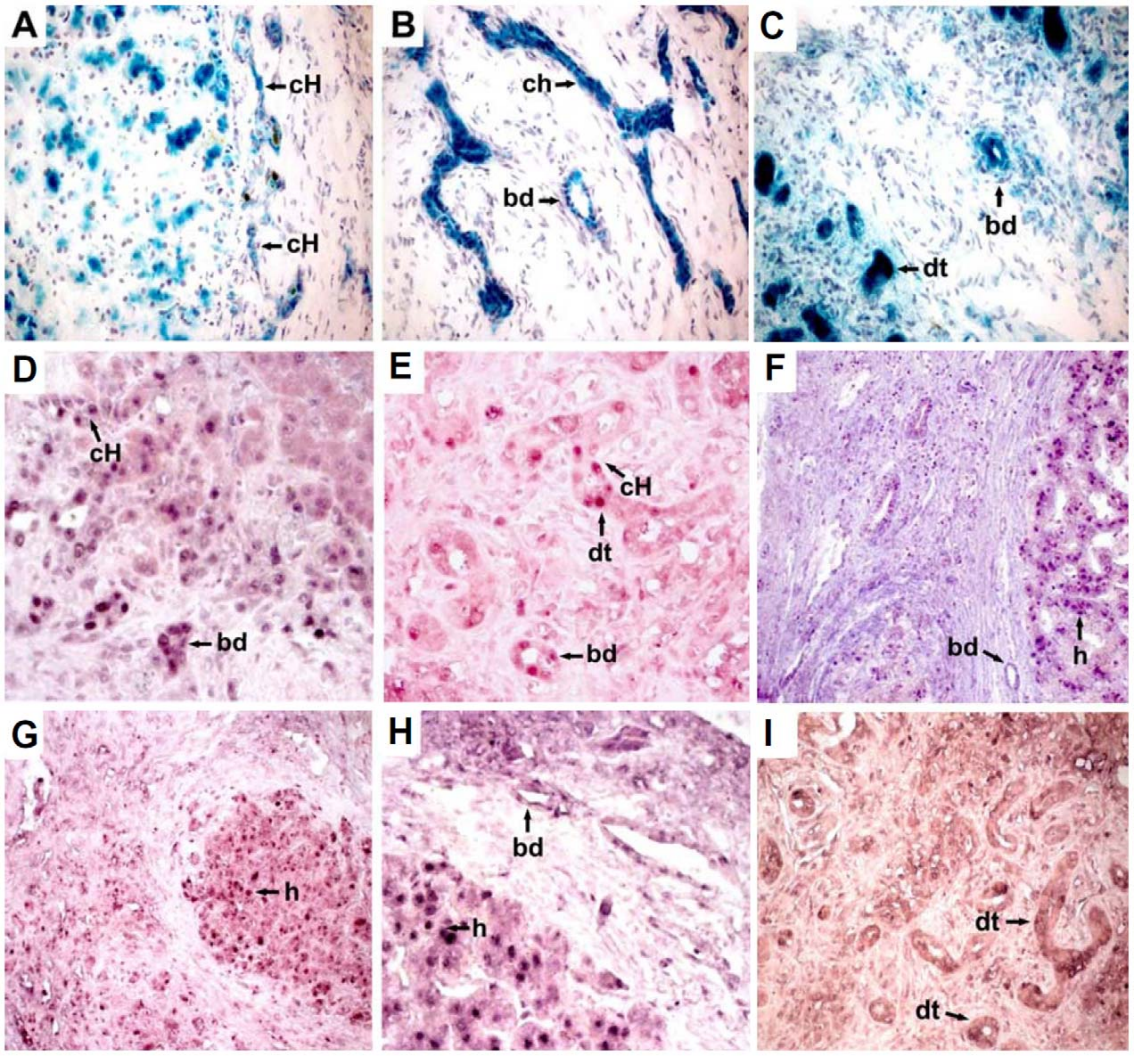
Representative images of senescence associated marker (β-galactosidase) and cell cycle markers (p16^INK4a^, p21^cip1^ and p53). SA-βgal expression (turquoise blue) in fresh liver sections of the recipients with end stage liver disease. **(A–B)** Intense SA-βgal in canals of Hering (cH), interlobular bile ducts (bd) and cholangioles (ch) in a patient with biliary atresia. (case 2) Magnification: 20×, 60×. **(C)** Ductular transformation of hepatocytes (dt) exhibiting SA-βgal in a patient with fulminant hepatic failure (case 4). Magnification: 40×. **(D–I)** Nuclear staining in formalin fixed paraffin embedded livers with end stage liver disease. **(D)** p53 canals of Hering (cH) and interlobular bile ducts (bd) in a patient with biliary atresia (case 3). **(E)** Canals of Hering (cH), interlobular bile ducts (bd) and hepatocyte ductular transformation (dt) in a patient with acute liver failure (case 4). **(F)** Hepatocytes in the regenerative nodules (h) and in the bile duct in the fibrotic bands (bd) in a patient with tyrosinemia (case 1), Magnification: 40×. **(G)** p16^INK4a^ staining in the hepatocytes (h) inside a regenerative nodule (case 1). Magnification: 20×, **(H)** p21^cip1^, staining in bile duct (bd) in the fibrotic bands and in the hepatocytes (h) inside a regenerative nodule (case 1). Magnification: 40×. **(I)** p21^cip1^ staining in the nuclei of cells that aggregate to form ductular transformation structures (dt) (case 4). Magnification 40×.

*SA-βgal activity* was detected in all five explanted livers. Its amount and distribution were heterogeneous. In all cases, staining was present in the canals of Hering and interlobular biliary ducts (TABLE 1). In the children with liver cirrhosis additional staining occurred in the cholangioles, and hepatocytes surrounding the regenerative nodules (Fig. 1A–B). In the livers of children with fulminant hepatic failure, intense staining was observed in the areas of ductal transformation (Fig. 1C). No SA-βgal activity was evident in the fibrous septa. In the livers of the five normal donors (with normal histology) SA-β gal activity was not detected.

**Table 1:** SA-βgal expression in the liver of children with end stage hepatic disease (%).

Patient	Nodule Hepatocytes		Canals of Hering	Ductular Transformation	Interlobular bile ducts	Cholangioles
	Central	Periphery				
**1**	5	10	100	NO	100	100
**2**	30	50	60	NO	100	60
**3**	Neg	20	10	NO	NO	10
**4**	Col	Col	100	90	100	neg
**5**	neg	neg	100	50	100	NO

Col = collapsed.

NO = not observed.

neg = negative.

### p53

p53 staining had a similar distribution to that observed for SA-βgal activity, but was less common among the structures (TABLE 2). In the cirrhotic livers (Fig. 1D and F) p53 staining was found in the canals of Hering, interlobular bile ducts and the hepatocytes in the regenerative nodules. In the patients with fulminant hepatitis p53 was also found in the ductular transformation areas (Fig. 1E).

**Table 2:** Expression of senescence markers in the liver of children with end stage hepatic disease (%).

Patient	Nodule Hepatocytes						Canals of Hering			Ductular Transformation			Interlobular bile ducts			Cholangioles		
	Central			Periphery														
	p16	p21	p53	p16	p21	p53	p16	p21	p53	p16	p21	p53	p16	p21	p53	p16	p21	p53
**1**	85	80	75	85	80	75	100	100	70	NO	NO	NO	35	100	15	100	100	Neg
**2**	neg	neg	neg	neg	neg	10	neg	neg	30	NO	NO	NO	neg	neg	30	neg	neg	50
**3**	neg	neg	20	neg	neg	50	neg	neg	60	NO	NO	NO	neg	neg	30	neg	NO	30
**4**	Col	Col	Col	Col	Col	Col	100	neg	30	30	50	80	30	20	30	neg	neg	50
**5**	Col	Col	Col	Col	Col	Col	neg	neg	>10	50	neg	80	100	20	30	NO	NO	NO

Col = collapsed.

NO = not observed.

neg = negative.

### p16^INK4a^ and p21^cip1^

Staining for p16^INK4a^ and p21^cip1^ was positive in the explanted liver of the patient with tyrosinemia in the hepatocytes, the canals of Hering, cholangioles and interlobular bile ducts (Fig. 1G–H) In the livers with fulminant hepatitis, p21^cip1^ staining occurred in the areas of ductal transformation and in the interlobular bile ducts (Fig. 1I; TABLE 2).

## Discussion

Cellular senescence is usually considered an attribute of aging. Its presence in livers of young children with end stage hepatic disease, characterized by SA- βgal activity accompanied by changes in the classical senescence pathway encompassing activation of p53, p21^cip1^ and p16^INK4a^, as found in the present study, support the concept that the loss of replicative capacity of the hepatocytes is related to liver damage.

Senescence markers were found for the most part in the Hering canals and the interlobular biliary ductules independent of the etiology of the underlying disease. All five children had an increased number of senescent cells accompanied with the expression of p53, reinforcing the concept of p53 as the major initiator of the senescence and a key regulator of DNA damage responses.

In the three cases with liver cirrhosis, the senescent hepatocytes were found predominantly around the regenerative nodules or inside of them (in lesser proportion) and absent in the fibrous septa. This distribution is similar to patterns previously described in adults [6]. In the two children with fulminant hepatic failure, senescent cells were displayed mainly at the site of the ductular transformation of the hepatocytes plate. It is now accepted that the atypical ductular reaction observed in livers with sub-massive hepatitis represents the proliferation of hepatic progenitor cells, similar to rat oval cells and are able to differentiate towards the biliary and the hepatocytic lineage [23]–[24]. Whether the ductular reaction observed in this study in the two children with fulminant hepatic failure represents an activated stem cell response or biliary metaplasia of cholestatic injured hepatocytes is not clear. When we used an antibody directed to CK7 (as a marker of biliary epithelium), approximately 60% of the staining was present in the tubular structures and all the staining was found in cells located in the canals of Hering (data not shown). The latter represent the anatomic and physiologic link between the intralobular canalicular system and the terminal branches of the biliary tree, suggesting that hepatocytes could have differentiated into biliary cells that aggregate to form the ductular structures [25] Transplantation of rat hepatocytes into a syngeneic rat spleen results in the appearance of CK 7 positive biliary cells that form ductules [26]. p21 is recognized as a valuable check point for evaluating proliferative activity/proliferation arrest of hepatocytes. In the case of fulminant hepatitis were more than 50% of hepatocytes where lost, p21 was found to be expressed in the ductular area where regeneration and hepatic progenitor cells probably fail in time to activate indicating disease severity. On the other hand the upregulated expression of p21 in the hepatocytes located in the regenerative nodules and its periphary in the case of tyrosinemia suggest the inhibition of cell proliferation and advance necrosis.

Overall, our findings point towards the equilibrium in the control of self-renewal and multilineage differentiation capacities of regenerative cells. For once the decline in the regenerative capacity and changes in number of functional stem/progenitor cells which at the same time may increase the risk to develop associated diseases and on the other the accumulation of genetic alterations leading to changes in regulation of numerous tumor suppressor genes. These processes in term could become be more relevant if deregulated signals from an aged niche, so as those of a small admixture of non dividing senescent cells could contribute to their dysfunctions or loss, which could account for some of the observed differences in time (chronic vs acute) of the patients herein reported. In order to further support the hypothesis that these senescence markers correlate with disease progression, it will be necessary to design studies including disease and age matched controls and to explore in more detail the possible mechanisms involved.

## Methods

### Patients

The study includes 5 consecutive patients aged three years or less with end stage liver disease due to tyrosinemia (n = 1) biliary atresia (n = 2) fulminant hepatitis (n = 2) who underwent liver transplantation at Mexico City's Children's Hospital (Hospital Infantil de Mexico). Complete clinical charts and pre-transplant liver biopsies were available for each case. The patients with liver cirrhosis due to tyrosinemia and biliary atresia had evidence of liver disease from the early days after being born. The patients with acute liver failure, one corresponded to an autoimmune etiology of seven months of progression with 30 days of acute liver failure and the other had nine days of disease progression of unknown etiology. The two patients with biliary atresia and liver cirrhosis had portal hypertension and esophageal bleeding. The two patients with acute liver failure have severe encephalopathy. Three patients received a liver allograft from living relatives and two from deceased donors. The surgical procedure and sample collections were described in detail to the patients' parents and appropriate signed informed consent was obtained. The study was approved by the institutional review board of the Hospital Infantil de Mexico.

### Explanted liver analyses

Sections from formalin fixed paraffin-embedded liver tissue were stained with haematoxylin and eosin, Masson's trichrome, PAS, PAS-diastase, modified orcein, reticulin Perl's iron, immunohistochemistry and special staining when necessary.

### SA-βgal Activity

Fresh tissue samples were used to detect activity of β-galactosidase at pH of 6.0 as marker of senescence. Surgical specimens were frozen immediately in OCT, cut into 5-µm sections and fixed with glutaraldehyde at 0.2% for 10 minutes. After washing with lacZ at room temperature the specimens were stained for 14–18 hrs at 37°C with fresh galactosidase staining solution. The solution contained 1 mg/ml 5-bromo-4-chloro-3-indolyl-β-D-galactoside (X-gal) (*Sigma-Aldrich, St Louis, MO, USA*). After staining, the specimens were washed twice with PBS. The specimens were counterstained with hematoxilin staining solution for one minute and washed twice in PBS. The cells with SA-βgal activity showed a blue turquoise staining in the cytoplasm.

### Immunohistochemistry

Deparaffinized tissue was microwave heated to retrieve antigen and reacted with full-length monoclonal antibodies to p53 (*Sigma-Aldrich, St Louis, MO, USA*) at a dilution of 1∶50, p21^cip1^ at a dilution of 1∶25 and p16^INK4a^ at a dilution of 1∶75, (*Santa Cruz Biotechnology, Santa Cruz, CA*) and incubated at 4°C overnight. A secondary antibody coupled with alkaline phosphatase was used to expose the reaction to the monoclonal antibodies; NBT/BCIP substrate was used as a chromogen. Carcinomatous colon tissue served as positive controls. Negative control slides were reacted with a non related antibody to *Aspergillus niger* (*Dako Cytomation, Glostrup, Denmark*) under similar conditions. Percentage of positive nuclear staining either weak or strong was noted. Quantification was performed by manual counting on 10 representative fields by two independent observers and photographed with a Nikon Microphot-FXA microscope. The mean of the scores counted by each of the observers was used as expression score.

An indirect immunohistochemical technique was utilized to stain for CK7 (*Dako Cytomation, Glostrup, Denmark*), employing a secondary antibody labeled with peroxidase and diaminobenzidine as a chromogen.
