# Correction: Cellular Senescence in Livers from Children with End Stage Liver Disease

**DOI:** 10.1371/annotation/6082f3f8-2b92-42a2-8d6f-b9210d2f25bf

**Published:** 2010-04-28

**Authors:** Gabriela Gutierrez-Reyes, Maria del Carmen Garcia de Leon, Gustavo Varela-Fascinetto, Pedro Valencia, Ruy Pérez Tamayo, Claudia Gonzalez Rosado, Blanca Farfan Labonne, Norma Morales Rochilin, Rosalinda Martinez Garcia, Jonathan Aguirre Valadez, Gabriela Togno Latour, Dana Lau Corona, Guillermo Robles Diaz, Albert Zlotnik, David Kershenobich

Affiliation 1 is incomplete. It should read: 1. Department of Experimental Medicine, School of Medicine, Universidad Nacional Autónoma de México (UNAM), Hospital General de México, Mexico City, Mexico.

